# Phthisis, or Pulmonary Tuberculosis

**Published:** 1898-06

**Authors:** J. L. Campbell

**Affiliations:** Atlanta, Ga., Demonstrator of Anatomy and Bacteriology, Atlanta Medical College


### PHTHISIS, OR PULMONARY TUBERCULOSIS.

BY J. L. CAMPBELL, M.D., ATLANTA, GA.,

Demonstrator of Anatomy and Bacteriology, Atlanta Medical College.

Tuberculosis is an infectious disease caused by Koch's bacillus tuberculosis. Authorities differ in their classification of the clinical form. The classification of Tyson and Osler seems to be best. They arrange it in three forms, viz.: 1st. Acute pneumonic phthisis. 2d. Chronic ulcerative phthisis. 3d. Fibroid phthisis.

*Etiology.*—The disease is caused by the bacillus tuberculosis, first demonstrated by Koch in 1882. He found the bacillus in the sputum of tubercular patients and was able to reproduce the disease in susceptible animals by inoculation with pure culture. Heredity plays an important part. Some authorities claim, among them Mr. Jonathan Hutchinson, that the bacillus is conveyed directly from the parent to the child, and in the tissues of the child remains latent until some favorable opportunity presents for its development. A better theory, however, is that children of tubercular parents inherit lungs that are better suited for the growth of tubercle bacillus. The bacilli, finding in these a suitable soil, develop, while in others they are thrown off in the expectoration. Phthisis also develops as one of the sequelæ of typhoid fever, pneumonia, influenza, and other acute febrile diseases.

This is the most wide-spread disease known to mankind. No nation, class or clime is exempt. It attacks all alike. It is more prevalent in the "slums" and poorer districts of our large cities than in the country. Here, as is the case with other infectious diseases, the surroundings are more suitable for its development. There are two modes of infection, one by the blood-vessels and lymphatics, and the other by inhalation. The sputum which contains the bacilli is dried and powdered into dust; in this the bacilli retain their vitality for weeks. This is a probable explanation of the fact that chambermaids and porters are so frequently infected with tuberculosis.

We will only consider chronic ulcerative phthisis. To this class belongs the great majority of cases of consumption. At first there may be a pure tubercular infection, but later the infection becomes mixed, and many of the most prominent symptoms are due to this septic infection. (Osler.)

*Morbid Anatomy.*—The disease may be divided into three stages, as in pneumonia, except in the third stage, instead of resolution and return to the normal, we have caseation and ulceration of the involved area. Lesions are located at the apices nearer to the posterior surface of the lung, or about an inch or a little more below the extreme apex, so that the first physical signs are obtained in the suprascapular fossa, or just below the middle of the clavicle. The extension is downward, probably due to the inhalation of the virus. Of the primary lesions in 427 apex cases collected by Osler, 172 were in the right apex, 130 in the left, 111 in both.

The lesion does not always occur at the apex. Dr. Percy Kidd, of the London Hospital, found the primary lesion occurred at the base in about one to five hundred cases. In Osler's cases the proportion was somewhat greater.

If the bacilli enter the lungs by inhalation they are carried to the smallest bronchial tubes, where they lodge, and as they begin to multiply they cause a proliferation of the connective tissue cells and epithelium; this continues until a nodule is formed. This is surrounded for a variable distance by an area of tubercular broncho-pneumonia. These nodules begin to caseate in the center; this caseous material liquefies and may break through into a bronchial tube. This pus or liquid is carried by the respiration into other parts of the lungs and new areas of infection result. At the point of the primary nodule a process of ulceration begins, which becomes infected with septic cocci. These nodules may caseate and become encysted and remain in the lung as caseous nodules. Calcification from deposit of lime salts may result in the "lung stone" frequently found at autopsies.

Cavities which have been rapidly formed will have soft, yielding walls. These are usually situated near the surface, and are most likely to break externally and give pneumothorax. Where the cavity results from caseation of a group of nodules, the walls are hard and well formed before the rupture takes place. There is little tendency for such cavities to increase in size. Cavities rarely heal. Scars are sometimes seen which are thought to be healed cavities, but we are not sure that this is the case.

There is usually a pleurisy over the affected area, the bronchial glands may be involved, the larynx is frequently the seat of tubercular ulcers, the vocal cords and epiglottis may be more or less affected. The tongue, gums and roof of the mouth were so involved in a case I saw, that it was with the utmost difficulty that the patient was able to take food. Other organs, the spleen, liver, kidneys and mesenteric glands, intestines and brains may be also affected. Frequently we find amyloid changes taking place in the liver, kidneys, and spleen. Fatty infiltration is not infrequent. To intestinal tuberculosis is due the diarrhea that is frequently the closing scene in a case of phthisis.

*Symptoms.*—The mode of onset varies; there may be symptoms of dyspepsia with loss of appetite, loss of flesh and general anemic appearance. It may simulate malaria and be mistaken for it. Hemorrhage may be the first indication of the diseased lung or a slight huskiness of the voice may be the first thing noticed. The patient however generally comes to us with a slight cough, dating from some cold which he imagines was neglected; the cough grows worse, appetite diminishes, he is losing flesh and probably has night sweats.

Pain in the chest is due to the accompanying pleuritis. The pain may be a troublesome symptom or may occur during paroxysms of coughing. It is usually felt beneath the scapulæ or at the apex of the lung.

A cough is nearly always present, at first dry and hacking. As the disease progresses there is a glairy muco-purulent expectoration. When cavities exist there is spasmodic coughing occurring more frequently in the early morning or after a rest in bed during the day. The sputum may vary in amount even in cases where there is extensive involvement. There may be scarcely any expectoration. At first the sputum is catarrhal in character and glairy in appearance; later, when breaking down occurs, it is more of a purulent character and small grayish or greenish masses are seen; these are almost pathognomonic. In them we should find the bacilli. The bacilli are not found as a rule in the early stages, as there is generally no destruction of tissue and the sputum is only mucous in character.

To examine the sputum pour it out in a large dish and select one of the whitish masses and spread it evenly on a well cleaned cover-slip, dry slowly over the flame of a Bunsen burner, then fix it by passing it three times slowly through the flame. Stain with carbol fuchsin or aniline water gentian violet. To stain with either of these pour a few drops on the cover-slip and hold it over the flame until it begins to boil, taking care that the cover-slip does not dry. Then wash with water, decolorize with twenty-five per cent. sulphuric acid, wash again with water and counter-stain with methylin blue. The bacilli will be seen as red rods three or four mm. in length and from three-tenths to five-tenths mm. in diameter. They may be slightly curved or take the stain irregularly. The clear spots are supposed to be spores. The bacilli may appear singly or in clumps of three or four. When found it is proof positive that the patient has tuberculosis. Other microorganisms, especially the pus cocci, are constantly present; blood may also be present in variable amounts as well as the bronchial elastic tissue. Hemoptysis may be present early from the erosion of a vessel by an ulcer, or it may occur later from the rupture of an aneurism into a cavity. There is not as a usual thing any marked dyspnea or rapidity of breathing, although considerable amount of lung tissue may be involved.

*General Symptoms.*—Fever is the most important and is one of the most valuable diagnostic points; there is nearly always a rise of from one-half to one and one-half degrees in the afternoon, which usually occurs between two and six o'clock. As the disease progresses the temperature may increase three or four degrees in the afternoon and one or two in the morning; this is partly due to mixed infection. The types vary; it may be entirely remittent even when there is great involvement of the lung and in rare cases is absent. There may be a subnormal temperature in the morning with an afternoon rise to 104 and 105. Sweating is most troublesome and extremely exhausting.

Pulse is increased in frequency and Page says it is a most important symptom for early diagnosis. The pulse is usually full, soft and easily compressible. The superficial veins are distinct, owing to absorption of adipose tissue beneath the skin. Complexion is sallow, skin is waxy in appearance and there may be some jaundice in advanced cases.

*Physical Signs.*—On inspection there may be some peculiar appearance about the chest of tubercular patients; the wing-like appearance of the scapulæ has often been described, as is also the antero-posterior narrowing of the chest. So far as expansion is concerned there is no marked change in the first stage, but as the disease progresses there is flattening and loss of expansion over the affected part. In cases where there is much thickening of the pleura one shoulder may be lower than the other. Over cavities of considerable size there will be marked flattening.

*Palpation.*—The tactile fremitus will be exaggerated over the affected part in the first stage, markedly increased as consolidation takes place; over cavities it will be increased if there is free communication with the trachea, but if the bronchus leading into the cavity be plugged with mucus it will be diminished.

*Percussion.*—The diseased area will give dullness varying with the amount of exudation from normal resonance to complete dullness where there is consolidation. Where cavities exist the percussion note varies with the depth of the cavity, elasticity or inelasticity of its walls.

*Auscultation* is by far the most important step in making a diagnosis; in the beginning of the disease even before there is dullness or increased tactile fremitus, you will be able to hear harsh, prolonged expiration, with a few fine moist râles; when this is heard in one or the other apex we should suspect phthisis. It must be borne in mind that in the right apex the respiratory murmur is more pronounced, and expiration is prolonged even in health. As the disease progresses these signs become more marked. The râles increase in number and the breathing approaches more and more a bronchial character until when consolidation has taken place there is pure bronchial breathing. Over cavities the breath-sounds differ according to the size, the elasticity of the walls and the amount of fluid contained in the cavities. Vocal resonance is increased as the disease progresses and is one of the best means of locating the diseased area. Vocal resonance is best appreciated when the patient whispers. We can distinctly hear the whispered word over the inflamed lung unless there is some plugging up of the bronchial tubes, while over the healthy lung the sound is more diffused.

*Diagnosis.*—Phthisis is usually located in the apex of one lung, while bronchitis is bilateral and has no tendency to consolidation. Pleurisy with an effusion may be mistaken for phthisis, but the line of dullness will be changed with the position of the patient. The tactile fremitus and the respiratory murmurs will be diminished or entirely absent.

As we have already stated, the bacilli are frequently absent in the early stage; but for a sure and comparatively safe plan of diagnosis, the use of Koch's tuberculin as recommended by Dr. J. Petruschky, of Dantzic, first the hypodermic injection of such quantities, one mg. of the tuberculin, as will give a moderate but sure reaction; then to be sure that the fever did not arise from some other cause, a second and third injection of a larger dose is to be given. If the reaction occurs after these injections the diagnosis is sure, as healthy individuals can take without the slightest reaction as much as ten to fifteen mg. of the lymph.

*Treatment.*—Place your patients where they can have the best hygienic surroundings possible, with plenty of sunlight and fresh air, moderate exercise and frequent baths, warm but not heavy clothing. Give them a good reconstituent tonic, light stimulants, and above all good food. Cough mixtures and expectorants do more harm than good. If the cough is troublesome we give tr. opii deod. ten drops, spts. ammon. arom. q.s. one dram. Give every four to six hours. Cod-liver oil is excellent when it is well borne.

If the case is favorable—*i. e.* were the amount of lung tissue involved is limited and the evening temperature does not exceed 100° F.—Koch's tuberculin is the ideal treatment, given in one-tenth mg. doses every alternate day. This may at first give a slight reaction. If on the following day the temperature is more than one degree above the normal, do not repeat the dose until the temperature has subsided. In only one of the cases I have treated in this manner did I get any troublesome reaction. On the second day after the first injection the patient had a slight chill, with a temperature of 102. Increased cough and profuse expectoration. This subsided in about two or three days and the treatment was continued.

At this time the patient is improving, his cough and expectoration has almost disappeared, he has no afternoon temperature and is gaining some flesh.

34½ *Peachtree St.*

